# Symptom-based pharmacotherapy for neuropathic pain related to spinal disorders: results from a patient-based assessment

**DOI:** 10.1038/s41598-022-11345-y

**Published:** 2022-05-03

**Authors:** Hideaki Nakajima, Shuji Watanabe, Kazuya Honjoh, Arisa Kubota, Akihiko Matsumine

**Affiliations:** Department of Orthopaedics and Rehabilitation Medicine, Faculty of Medical Sciences, University of Fukui, 23-3 Matsuoka Shimoaizuki, Eiheiji-cho, Yoshida-gun, Fukui, 910-1193 Japan

**Keywords:** Neurological disorders, Drug regulation, Therapeutics, Outcomes research, Orthopaedics

## Abstract

Existing guidelines advocate an updated therapeutic algorithm for chronic neuropathic pain (NeP), but pharmacotherapeutic management should be individualized to pain phenotypes to achieve higher efficacy. This study was aimed to evaluate the efficacy of medications, based on NeP phenotypes, and to propose symptom-based pharmacotherapy. This retrospective study was enrolled 265 outpatients with chronic NeP related to spinal disorders. The patients were classified into three groups: spinal cord-related pain, radicular pain, and cauda equina syndrome. Data were obtained from patient-based questionnaires using Neuropathic Pain Symptom Inventory (NPSI) and the Brief Scale for Psychiatric Problems in Orthopaedic Patients, and from clinical information. The proportions of patients with ≥ 30% and ≥ 50% reduction in NPSI score for each pain subtype (spontaneous pain, paroxysmal pain, evoked pain, and paresthesia/dysesthesia) and drugs were evaluated. The pain reduction rate was significantly lower in patients with spinal cord-related pain, especially for paresthesia/dysesthesia. For spinal cord-related pain, duloxetine and neurotropin had insufficient analgesic effects, whereas mirogabalin was the most effective. Pregabalin or mirogabalin for radicular pain and duloxetine for cauda equina syndrome are recommended in cases of insufficient analgesic effects with neurotropin. The findings could contribute to better strategies for symptom-based pharmacotherapeutic management.

## Introduction

Neuropathic pain (NeP) commonly develops after interventional procedures that affect somatosensory signaling in the peripheral or central nervous system^1,2^. The associated significant reduction in the health-related quality of life and increase in economic costs make NeP treatment critical^3–5^. A cross-sectional study revealed that more than half (53.3%) of the patients with spinal disorders had NeP using screening questionnaire to identify potential patients with NeP, and the highest incidence (77%) of NeP was among patients with cervical spondylotic myelopathy, followed by ligament ossification (75%), spine/spinal cord injury (65%), nerve root damage (57%), and lumbar spinal stenosis (56%)^6^. A major concern for our globally aging society is that spinal disorders occur more frequently in aged individuals. Moreover, an epidemiological study indicated that a rate 65.0–78.8% patients with NeP experience chronic pain^7^. Although further validation of NeP screening questionnaire is needed for epidemiological purposes^8^, it becomes important to pay particular attention to improve pain recognition and management in NeP related to spinal disorders, particularly in older adults.

Guidelines and consensus statements for treatment of NeP have been published worldwide^9–13^. The NeP pharmacotherapy algorithm in Japan recommends pregabalin, mirogabalin, and duloxetine as the first-line drugs, and neurotropin (an extract from inflamed cutaneous tissue of rabbits inoculated with the vaccinia virus) and tramadol as the second-line drugs. However, these statements apply to NeP in general, without considering underlying causes and differences in the types of NeP. It is difficult to predict and assess the treatment response in cases of NeP because the same underlying disease may manifest differently in different patients or because different diseases may present with similar clinical features^14,15^. Prescribing doctors currently have the discretion regarding which drug to use. Previous studies recommend symptom-based and/or mechanism-based treatment because pharmacological treatment efficacy is associated with NeP phenotype and severity^16–19^. This indicates the need for individualized management of NeP through prediction of treatment responses based on individual pain phenotypes.

NeP classification based on different triggering stimuli and the use of multidisciplinary approaches may help us better understand the underlying mechanisms of pain in patients and facilitate the development of appropriate pain management protocols. Therefore, this study was conducted to investigate the type and severity of NeP, including the degree of deterioration of psychiatric factors, by using patients-based questionnaires, and to assess the drug-specific effectiveness of treatment, based on the type of pain.

## Results

### Clinical characteristics

Table 1 summarizes the clinical characteristics of the 265 patients. The patients most frequently had cervical spondylosis (n = 60; 22.6%), cervical spine OPLL (n = 38; 14.3%), cervical spinal cord injury (n = 22; 8.3%), and LSS (n = 145; 54.7%). Spinal surgery was performed in 40% patients, and 29.8% patients had comorbid diabetes. NeP was classified into spinal cord-related pain (n = 87; 32.8%), radicular pain (n = 96; 36.2%), and cauda equina syndrome (n = 82; 30.9%). Of these 265 patients, 109 patients had taken pregabalin, 63 patients had taken mirogabalin, 54 patients had taken duloxetine, and 39 patients had taken neurotropin. Tramadol was used to treat patients experiencing poor pain reduction effects when taking a single neurotrophic pain medication. The median daily prescription doses were 150 mg for pregabalin, 20 mg for mirogabalin, 40 mg for duloxetine, 16 units for neurotropin, and 100 mg for tramadol. In the duloxetine group, the NPSI score remained significantly higher, even with the use of tramadol (Table 2).

**Table 1:** Summary data for 265 patients.

Age, years, mean ± SD	68.9±12.6
Gender, male (%)/female (%)	152 (57.4)/113 (42.6)
**Underlying disorders, n (%)**	
Cervical spondylosis	60 (22.6)
Ossification of longitudinal ligament	38 (14.3)
Spinal cord injury	22 (8.3)
Lumbar spinal canal stenosis	145 (54.7)
Past spine operation, n (%)	106 (40.0)
Comorbidity of diabetes, n (%)	79 (29.8)
**Neurological symptoms, n (%)**	
Spinal cord-related pain	87 (32.8)
Radicular pain	96 (36.2)
Cauda equina syndrome	82 (30.9)
**Drug, n (number of patients taking concomitant tramadol)**	
Pregabalin	109 (40)
Mirogabalin	63 (20)
Duloxetine	54 (8)
Neurotropin	39 (0)
**Median daily prescription dose (approved dose), mg or units**	
Pregabalin	150 (300)
Mirogabalin	20 (30)
Duloxetine	40 (40 or 60)
Neurotropin	16 (16)
Tramadol	100 (150)

**Table 2:** Differences in NPSI scores with and without the concomitant use of tramadol.

	NPSI score		p
	Tramadol (+)	Tramadol (−)	
Pregabalin	15.6±10.9	14.2±8.8	0.56
Mirogabalin	18.3±10.9	14.0±8.6	0.20
Duloxetine	25.5±11.2	14.1±7.7	0.025*

*NPSI* Neuropathic Pain Symptom Inventory.

*p<0.05.

### Association of treatment efficacy with NeP Type

To assess the drug-specific effectiveness of treatment based on neurological symptoms, the proportions of patients with ≥ 30% reduction or with ≥ 50% reduction in Neuropathic Pain Symptom Inventory (NPSI) score or subscore from baseline to follow-up were determined.

**Figure 1:**
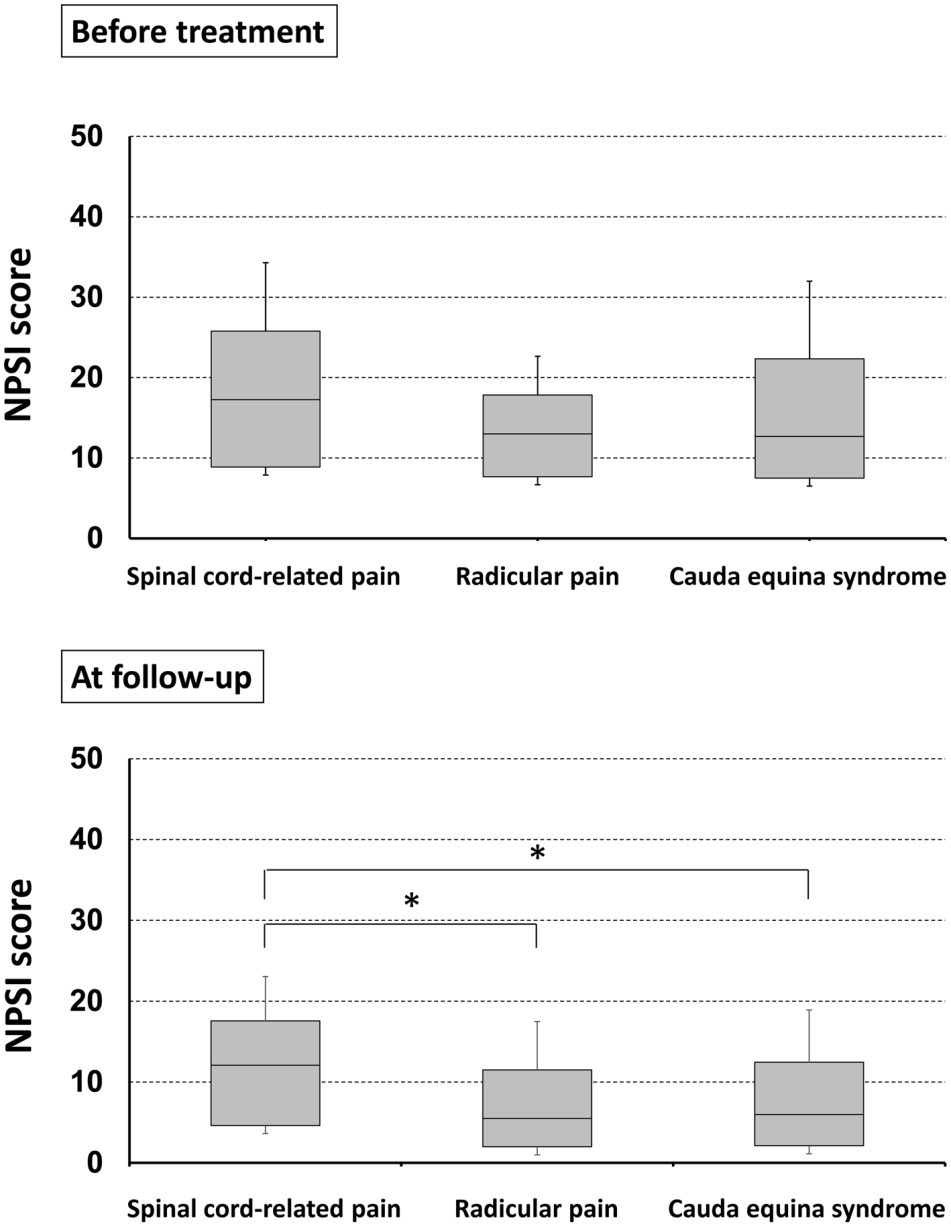
Neuropathic pain system inventory (NPSI) scores in patients with spinal cord-related pain, radicular pain, and cauda equina syndrome. Moderate pain remained at 12.5 points, even at follow-up, in patients with spinal cord-pain. *p<0.05. *NSPI* Neuropathic Pain Symptom Inventory.

Figure 1 shows the NPSI scores at baseline and at follow-up in patients with spinal cord-related pain, radicular pain, and cauda equina syndrome. The NPSI scores at baseline were higher in patients with spinal cord-related pain, but the difference was not significant (p = 0.066). However, the moderate pain score remained at 12.5 points, even at follow-up, in patients with spinal cord pain. Pain reduction rates based on the NPSI scores and subscores and responder rates in patients with spinal cord-related pain, radicular pain, and cauda equina syndrome are shown in Table 3. The baseline NPSI item subscores did not differ significantly between the three groups, but patients with spinal cord-related pain had significantly lower pain reduction rates for evoked pain and paresthesia/dysesthesia than those with radicular pain and cauda equina syndrome and for spontaneous pain (i.e., superficial and deep pain) than for radicular pain. Patients with spinal cord-related pain had significantly lower total response rates than patients with radicular pain (p < 0.01). The 30% and 50% responder rates were also significantly lower in patients with spinal cord-related pain (41.4% and 16.1%, respectively).

**Table 3:** NPSI subscores and response rates in patients with spinal cord-related pain, radicular pain, and cauda equina syndrome.

	Spinal cord-related pain	Radicular pain	Cauda equina syndrome	p
Number of patients	87	96	82	
**NPSI subscore**				
Superficial pain	31.0 ± 27.2^‡^	46.9 ± 31.0	35.7 ± 21.3	0.035*
Deep pain	25.7 ± 22.0^‡^	42.9 ± 27.9	36.3 ± 24.7	0.024*
Paroxysmal pain	37.4 ± 30.0	38.9 ± 23.8	32.2 ± 26.8	0.74
Evoked pain	15.8 ± 17.8^†^	40.0 ± 25.9	35.0 ± 22.2	< 0.01*
Paresthesia/dysesthesia	21.0 ± 20.9^†^	41.6 ± 23.9	35.8 ± 24.5	< 0.01*
Total pain reduction rate	25.2 ± 22.0^‡^	42.2 ± 26.0	33.6 ± 20.0	< 0.01*
**Responder**				
30% Responder	36/87 (41.4)^‡^	66/96 (68.8)	52/82 (63.4)	< 0.01*
50% Responder	14/87 (16.1)^‡^	34/96 (35.4)	31/82 (37.8)	< 0.01*

Data are shown as mean ± SD.

*NPSI* Neuropathic Pain Symptom Inventory.

*Significant differences among groups.

^†^p<0.05, compared to patients with radicular pain and cauda equina syndrome.

^‡^p<0.05, compared to patients with radicular pain.

The drug-specific NPSI scores and subscores at baseline and at follow-up are shown in Table 4. No significant differences occurred in the response rate for each drug. However, some interesting trends were observed: (1) duloxetine and neurotropin would not be expected to have a significant effect on spinal cord-related pain (i.e., the 50% responder rate was 0%); (2) mirogabalin was most effective for patients with spinal cord-related pain syndrome, although the response rate was not high (50% responder rate, 29.2%); (3) for patients with less severe radicular pain and cauda equina syndrome, neurotropin would be expected to have pain reduction effects (i.e., the NPSI score at baseline was 11.8 ± 6.5 in responders and 18.8 ± 10.5 in nonresponders, p = 0.19); (4) duloxetine tends to be less effective than the other drugs in treating radicular pain (the 30% responder rate was 46.7%); and (5) some patients with cauda equina syndrome responded well to duloxetine (the 50% responder rate was 45.8%).

**Table 4:** The efficacy of each drug, evaluated by using the neurological symptoms of neuropathic pain related to spinal disorders.

	Drug				p
	Pregabalin	Mirogabalin	Duloxetine	Neurotropin	
Number of patients	109	63	54	39	
**Spinal cord-related pain**					
Base-line NPSI	16.1±9.1	18.2±11.4	17.2±9.0	18.3±13.1	0.99
Follow-up NPSI	12.4±9.4	13.1±9.3	14.4±9.8	15.7±7.9	0.81
30% Responder, n (%)	16/40 (40.0)	11/24 (45.8)	7/15 (46.7)	2/8 (25.0)	0.74
50% Responder, n (%)	7/40 (17.5)	7/24 (**29.2**)	0/15 (**0**)	0/8 (**0**)	0.055
**Radicular pain**					
Baseline NPSI	14.9±10.2	13.0±7.7	15.0±10.5	11.6±6.4	0.77
Follow-up NPSI	8.6±7.8	8.1±5.0	10.1±6.8	7.2±5.4	0.64
30% Responder, n (%)	32/45 (71.1)	15/20 (75.0)	7/15 (**46.7**)	12/16 (75.0)	0.27
50% Responder, n (%)	15/45 (33.3)	7/20 (35.0)	5/15 (33.3)	7/16 (43.8)	0.89
**Cauda equina syndrome**					
Baseline NPSI	15.6±11.4	16.6±10.1	17.7±10.3	12.7±5.5	0.68
Follow-up NPSI	11.5±8.2	11.3±8.1	11.8±8.3	6.5±3.8	0.30
30% Responder, n (%)	13/24 (54.2)	12/19 (63.2)	15/24 (62.5)	12/15 (80.0)	0.47
50% Responder, n (%)	6/24 (25.0)	7/19 (36.8)	11/24 (**45.8**)	7/15 (46.7)	0.41

Data are shown as the mean ± the standard deviation. Bold, notable results.

^⁑^p<0.01, compared to total patients with radicular pain and cauda equina syndrome.

*NPSI* Neuropathic Pain Symptom Inventory.

### Therapeutic effects of drugs, based on the characteristics of neuropathic pain

To assess the drug-specific changes for each pain phenotype, the pain reduction rates in the NPSI scores and subscores in patients with a NPSI score of ≥ 1 at baseline and at follow-up were analyzed. The pain reduction rate of duloxetine for patients with paresthesia/dysesthesia was lower than that of the other drugs. For evoked pain, the average pain reduction rate of duloxetine was below 30%, which was also lower than that of the other drugs, although the difference was not statistically significant (Table 5).

**Table 5:** The response rate of each drug for patients with an NPSI subscore ≥ 1.

	Pregabalin	Mirogabalin	Duloxetine	Neurotropin	p
**Superficial pain**					
Baseline NPSI subscore	5.2±2.5	5.4±2.8	5.9±2.6	4.2±1.7	0.11
Follow-up NPSI subscore	3.1±2.3	3.6±2.7	4.0±2.6	2.5±1.6	0.27
Response rate (%)	40.4±30.3	38.8±26.7	34.9±26.3	43.7±24.0	0.74
**Deep pain**					
Baseline NPSI subscore	6.6±2.2	5.5±2.0	6.6±2.3	6.1±1.5	0.41
Follow-up NPSI subscore	4.0±2.6	3.3±1.7	4.4±2.6	3.9±1.6	0.66
Response rate (%)	40.9±32.0	39.3±23.2	35.1±29.3	38.1±13.0	0.93
**Paroxysmal pain**					
Baseline NPSI subscore	4.3±2.3	4.5±2.5	4.0±2.3	3.8±2.1	0.75
Follow-up NPSI subscore	2.9±1.9	3.3±2.6	2.8±2.0	2.5±1.9	0.83
Response rate (%)	34.2±29.1	33.8±26.6	35.2±28.4	40.0±17.5	0.86
**Evoked pain**					
Baseline NPSI subscore	3.7±2.7	4.9±2.5	4.1±2.9	3.9±2.2	0.41
Follow-up NPSI subscore	2.7±2.4	3.6±2.5	3.2±2.6	2.6±2.1	0.44
Response rate (%)	32.0±27.1	31.5±25.5	**25.1±22.6**	38.8±20.8	0.40
**Paresthesia/dysesthesia**					
Baseline NPSI subscore	5.0±2.4	4.9±2.0	5.5±2.6	4.6±2.0	0.67
Follow-up NPSI subscore	3.5±2.3	3.4±2.1	4.0±2.4	2.7±1.8	0.10
Response rate (%)	32.0±27.4	32.5±28.2	**26.1±25.0**	45.7±19.3	0.013*
**Total**					
Baseline NPSI score	15.0±10.0	15.6±9.6	16.5±9.6	13.8±8.3	0.63
Follow-up NPSI score	10.1±8.2	10.5±7.6	11.7±8.0	8.5±6.4	0.29
Response rate (%)	33.9±27.1	34.6±21.7	30.7±24.8	42.3±18.8	0.091

Data are shown as mean ± SD. Bold, notable results.

*NPSI* Neuropathic Pain Symptom Inventory.

*p < 0.05.

### Association of patient background and BS-POP scores between the responders and nonresponders

A comparison of the patient background and BS-POP scores between responder and nonresponder is summarized in Table 6. No differences were observed in terms of age, gender, the proportion of patients with diabetes as the comorbidity. To assess the effect of psychiatric problems on treatment efficacy, the differences in BS-POP scores were analyzed. The rates of patient and doctor BS-POP scores (i.e., > 15 and > 10, respectively) did not differ significantly between responders (i.e., pain reduction rate ≥ 30%) and nonresponders (i.e., pain reduction rate < 30%). However, the total rates were high in both groups, and exceeded 40% (Table 6).

**Table 6:** Differences in patient background and the number of participants with a BS-POP score of ≥ 15 for patients or ≥ 10 for doctors between responders and nonresponders.

	Responder (n=154)	Nonresponder (n=111)	p
**Patient background**			
Age, years, mean ± SD	68.1±11.8	69.2±13.3	0.52
Gender, male (%)/female (%)	87 (56.5)/67 (43.5)	65 (58.6)/46 (41.4)	0.83
Comorbidity of diabetes, n (%)	43 (27.9)	36 (32.4)	0.51
**BS-POP (Pt ≥ 15 and Dr ≥ 10)**			
Pregabalin, n (%)	32/61 (52.5)	18/48 (37.5)	0.13
Mirogabalin, n (%)	16/38 (42.1)	17/25 (68.0)	0.071
Duloxetine, n (%)	17/29 (58.6)	14/25 (56.0)	1.00
Neurotropin, n (%)	9/26 (34.6)	6/13 (46.2)	0.51
Total, n (%)	69/154 (44.8)	45/111 (40.5)	0.53

Data are shown as the number of applicants/total number (%).

*BS-POP* Brief Scale for Psychiatric Problems in Orthopaedic Patients.

*p<0.05.

## Discussion

Existing guidelines advocate first- to third-line treatment in the updated therapeutic algorithm. However, we believe that the response rate can be improved if we determine which medications are more likely to be effective for which symptoms. The aim of the current real-world study, using patient-based assessments, was to investigate the effectiveness of treatment, based on the type of NeP related to spinal disorders, and to propose a symptom-based therapeutic algorithm, based on information about which medications are more likely to be effective for which symptoms. The major findings of our current research were: (1) patients with radicular pain and cauda equina syndrome commonly had spontaneous pain and paresthesia/dysesthesia, whereas patients with spinal cord-related pain prominently had more severe paresthesia/dysesthesia (especially tingling); (2) the pain reduction rate was significantly lower in patients with spinal cord-related pain, especially for paresthesia/dysesthesia, and the NPSI score at follow-up remained > 10. Our previous nationwide survey and multicenter cross-sectional study of spinal cord-related pain syndrome also showed similar characteristics of patients with spinal cord-related pain^20,21^; (3) duloxetine and neurotropin are not expected to have a significant effect on spinal cord-related pain, based on the lower response to paresthesia/dysesthesia. For these patients, mirogabalin may be recommended; (4) Neurotropin could be a first-line drug for radicular pain and cauda equina disorder as a drug with few serious adverse effects. Pregabalin or mirogabalin for radicular pain and duloxetine for cauda equina syndrome may be recommended for patients experiencing poor analgesic effects with neurotropin. These proposals may contribute to better strategies as symptom-based pharmacotherapeutic management for the relief of NeP related to spinal disorders.

Pregabalin inhibits the release of excitatory neurotransmitters by combining with the α_2_δ subunits of voltage-dependent calcium (Ca^2+^) channels in the central nervous system and, compared to a placebo, it has significant analgesic effects on postherpetic neuralgia^22^, diabetic neuropathy^23^, and pain after spinal cord injury^24^. Its effectiveness on depression and NeP-associated anxiety have also been demonstrated. Mirogabalin has a higher binding affinity for the α_2_δ subunits, and the dissociation rate is slower for the α_2_δ-1 subunit contributes to analgesic effects than for the α_2_δ-2 subunit contributes to undesirable central nervous system effects resulting in more sustained analgesia compared with traditional gabapentinoids^25,26^. Several randomized controlled trials (RCT) have verified the efficacy of mirogabalin for treating NeP such as postherpetic neuralgia and diabetic neuropathy^27,28^. In addition, an application for approval for central NeP (e.g., pain after spinal cord injury and cerebral infarction) is under review in Japan. In our study, pregabalin and mirogabalin each showed good efficacy with 30% responder rates > 70% in patients with radicular pain. An interesting finding is that, for patients experiencing spinal cord-related pain for whom NeP may be very difficult to treat, the 50% responder rate was higher in the mirogabalin group than in the pregabalin group. However, the median dose of both groups was smaller than approved doses used in the clinical trials.

The prescription status of oral analgesics, based on the findings of a large-scale prescription database in Japan^29^, revealed that many analgesics [e.g., pregabalin (median dose, 75 mg) and duloxetine (median dose, 20 mg)], were prescribed at lower doses than the approved doses. Prescribing an approved dose is an important factor in achieving a therapeutic effect, but the relatively high incidence of adverse effects such as dizziness and somnolence are also a substantial problem with both drugs^30,31^. The results of the current study may be because a therapeutic effect was easier to achieve with mirogabalin, which is easier to increase the dose, rather than because of the effect of the drug itself. However, we believe that the ease of attaining the approved dose is also an important factor when considering which drugs to choose. In addition, a previous study suggested that continued oral mirogabalin treatment increases the pain threshold over time^26^. A retrospective study indicated that mirogabalin is safe and effective for reducing NeP in patients who ceased treatment with pregabalin because of adverse events or lack of efficacy^32^.

Duloxetine acts on the serotonin and noradrenalin system, which is involved in the descending pain inhibitory system. Its analgesic effects are induced by inhibiting serotonin-noradrenaline reuptake. Some RCTs indicate that it has analgesic effects for diabetic neuropathy and chemotherapy-induced peripheral neuropathy^33–36^. Furthermore, case-series studies report analgesic effects on peripheral neuropathy accompanying multiple sclerosis and central poststroke pain^37,38^. Our results suggested that duloxetine is unlikely to have high efficacy for patients with spinal cord-related pain owing to its low efficacy in paresthesia/dysesthesia. However, another retrospective study indicated that duloxetine had therapeutic effect for postsurgical chronic myelopathic disorders of pain and numbness in the chronic stage after surgery^39^. For patients with radicular pain in this study, the 30% responder rate also tended to be lower than that of other drugs. However, an interesting finding was that the group of patients with cauda equina syndrome responded well to treatment (50% responder rate, 45.8%). In an animal study, the analgesic effects of pregabalin were weaker in the cauda equina compression model without an improvement in the walking distance than in the partial sciatic nerve ligation model; the expression of α_2_δ-1 subunit significantly increased only in the partial sciatic nerve ligation model and remained unchanged in the cauda equina compression model. However, duloxetine had analgesic effects in both models and improved walking distance in the cauda equina compression model^40^. The expression of the α_2_δ-1 subunit may be specific to the injury type. These experimental results seem to be in line with our clinical results, and this finding is important for considering symptom-based treatment for NeP.

Neurotropin contains nonprotein-type biologically active substances, which are extracted from the inflamed cutaneous tissue of rabbits inoculated with vaccinia virus. The analgesic effect of neurotropin occurs by activating the descending pain inhibitory system, exerting an anti-inflammatory action, inhibiting the release of excitatory neuropeptides, inhibiting the excitation of sympathetic nerves, improving blood flow, and exerting a neuroprotective action^41^. In addition to its analgesic effects, patients have very high tolerability to the drug with no serious adverse reactions. In our study, neurotropin had sufficient analgesic effects for radicular pain and cauda equina syndrome, especially for the patients without severe NeP; however, its effectiveness in treating spinal cord-related pain is not expected.

Tramadol acts as μ-opioid receptor agonist and as a serotonin-noradrenaline reuptake inhibitor. It exerts its analgesic effects by suppressing the ascending pain pathways and activating the descending pain inhibitory system. Previous RCTs indicate it has analgesic effects in patients with diabetic neuropathy^42^, postherpetic neuralgia^43^, and post-spinal cord injury^44^. Concomitant use of tramadol with another NeP medication may enhance analgesic effects because tramadol can prevent abnormal excitability of neuronal firing in ascending and descending pain pathways (e.g., the spinal cord and in brain lesions).

NeP can also markedly affect mental health, activities of daily life (ADLs), and physical conditions and function. The scores for all short form (SF)-36 subitems among patients with NeP were significantly lower than the national average scores^20^. Severe pain impairs employment and daily life in approximately 25% of patients^21^. Furthermore, chronic musculoskeletal pain was associated with future decline in ADL in a longitudinal study^45^. In drug-specific assessments, nonresponders were significantly more likely to have psychological problems in patients with taking mirogabalin, but no association between treatment efficacy and psychological factors was noted with the other drugs. The finding that more than 40% of patients (which included nonresponders and responders) had psychiatric problems at follow-up was a matter of concern, although our results suggested that the influence of psychiatric problems on treatment efficacy may be small.

**Figure 2:**
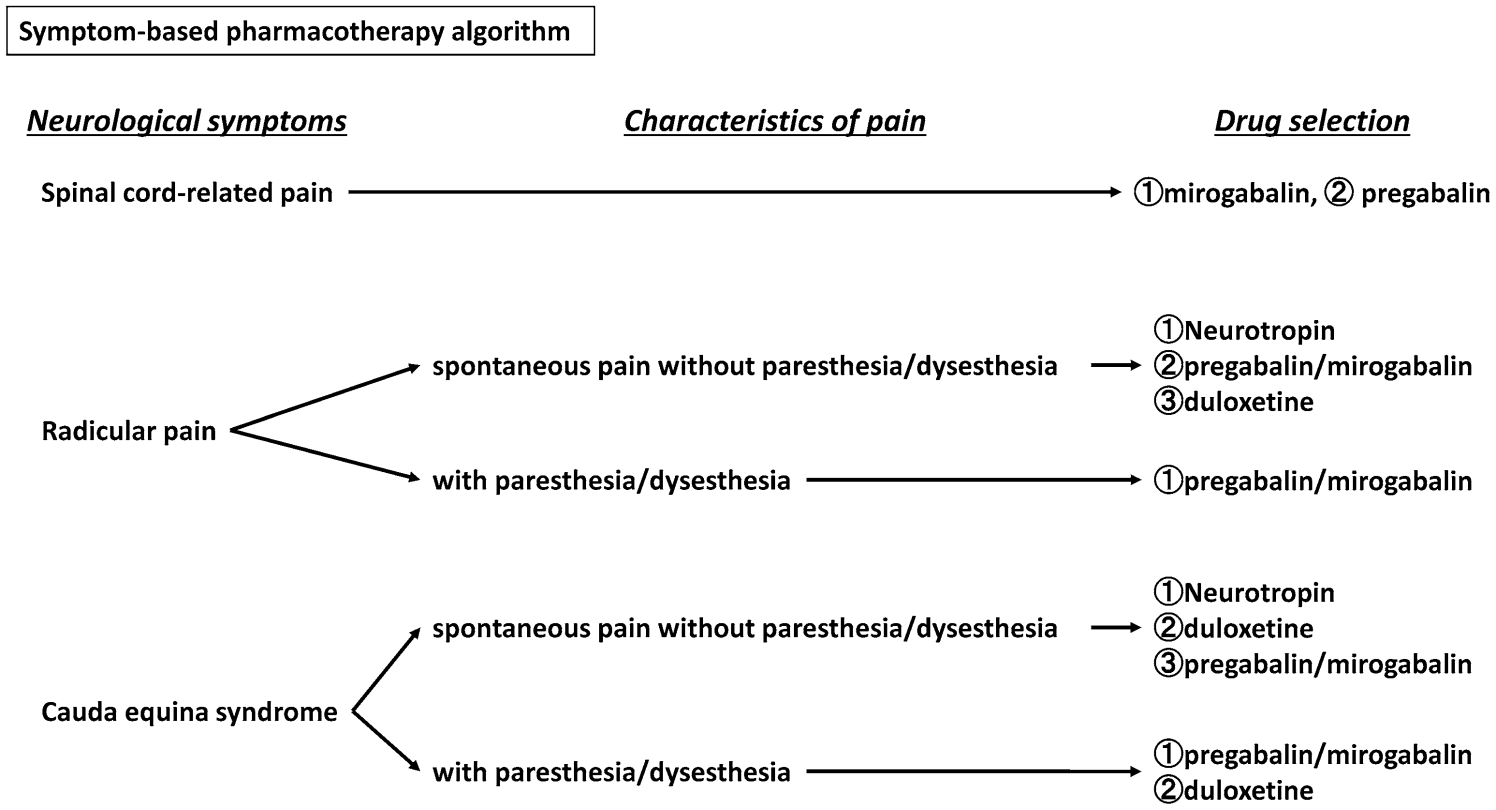
Symptom-based pharmacotherapy for patients with neuropathic pain related to spinal disorders. The drug should be chosen, based on the neurological symptoms and characteristics of the pain.

Based on the results of our study, we proposed a symptom-based pharmacotherapy algorithm as shown in Fig. 2. For patients with spinal cord-related pain, mirogabalin may be recommended as the first-line drug, and pregabalin as the second-line drug. For patients with radicular pain and cauda equina syndrome, neurotropin may be expected to have sufficient analgesic effects if NeP is not severe without paresthesia/dysesthesia. However, for these patients with paresthesia/dysesthesia, pregabalin or mirogabalin may be recommended as the first-line drug. For patients with cauda equina syndrome without paresthesia/dysesthesia, duloxetine could be recommended for patients who have a poor analgesic effect with neurotropin.

The retrospective, single-center design and the presence of possible confounding factors for which we were unable to adjust could be construed as some limitations of the current study. Our proposed symptom-based pharmacotherapy for NeP related to spinal disorders was inferred from patient-based assessments in the present study and require verification in a future study. Although it will be beneficial to conduct a larger prospective study to determine the significance of the results, the present study provides useful insights and guidance on the symptom-based therapeutic management of patients with NeP caused by spinal disorders.

## Methods

### Study design

This retrospective study included outpatients with chronic NeP related to spinal disorders. NeP is pain initiated or caused by a primary lesion or dysfunction in the nervous system diagnosed using the algorithm (grading system) formulated by Neuropathic Pain Special Interest Group (NeuPSIG) of International Association for the Study of Pain (IASP)^2^. These patients visited our hospital in 2020 and had taken a fixed dose of pregabalin, mirogabalin, duloxetine, or neurotropin for ≥ 3 months with or without concomitant tramadol. Patients taking multiple medications of the former four drugs were excluded. Chronic NeP associated with a spinal disorder was diagnosed, using the following criteria: (1) persistent pain for at least 3 months; (2) the presence of compressive lesions, confirmed by imaging and consistent with neurological findings; (3) poor response to nonsteroidal anti-inflammatory drugs; and (4) clinical confirmation of an absence of neurodegenerative disease, brain disease, history of chemotherapy, and peripheral nerve disorders (e.g., diabetic neuropathy, strangulated neuropathy)^18^. Patients with no NPSI score or no applicable items (no pain, numbness, and/or hyper- or hypoesthesia) at base-line were excluded from this study.

The patients were also classified into three groups, based on the type of neurological symptoms: (1) spinal cord-related pain, (2) radicular pain, and (3) cauda equina syndrome. As described previously^18,20,21^, spinal cord-related pain was defined as chronic NeP in patients with spinal cord-associated diseases [e.g., spinal cord injury, spinal myelopathy, and ossification of the posterior longitudinal ligament (OPLL)]. Radicular pain was defined as upper or lower extremity pain consistent with a neurological dominant region due to lumbar spinal canal stenosis (LSS) or cervical spondylosis. Cauda equina syndrome in patients with LSS was diagnosed, based on symptoms of neurogenic intermittent claudication or motor and/or sensory disturbance, including bladder dysfunction. Three senior spine surgeons conducted all neurological evaluations. Before filling out the questionnaire, written informed consent was obtained from each patient. The study protocol was approved by the Human Ethics Review Committee of Fukui University Medical Faculty, and it strictly followed the Clinical Research Guidelines of the Ministry of Health, Labor, and Welfare of the Japanese Government.

### Patient-based questionnaires

Two questionnaires were used in the study. The first questionnaire administered to patients was the Neuropathic Pain Symptom Inventory (NPSI)^19^. Within the NPSI scoring system, subscores were evaluated for burning (i.e., superficial) spontaneous pain, pressing (i.e., deep) spontaneous pain, paroxysmal pain, evoked pain, and paresthesia/dysesthesia (10 possible points for each subscale and a possible total of 50 points). The second questionnaire administered to patients was the Brief Scale for Psychiatric Problems in Orthopaedic Patients (BS-POP) (doctor and patient versions), which was used to evaluate psychiatric problems^46^.

### Outcome variables

Total NPSI score and NPSI subscore at baseline and at follow-up were evaluated to assess the treatment efficacy according to the neurological symptoms (spinal cord-related pain, radicular pain, and cauda equina syndrome), pain subtype (superficial spontaneous pain, deep spontaneous pain, paroxysmal pain, evoked pain, and paresthesia/dysesthesia) and selected drugs (pregabalin, mirogabalin, duloxetine, or neurotropin). The pain reduction rate (%) was calculated, as follows: [NPSI score (subscore) at baseline − NPSI score (subscore) at follow-up] × 100/NPSI score (subscore) at baseline. Treatment outcomes were also analyzed, based on the responder rate (i.e., the proportion of patients with ≥ 30% reduction or with ≥ 50% reduction in the NPSI score or subscore from baseline to follow-up)^18^. In addition, the BS-POP (doctor and patient versions) were evaluated to assess the degree of deterioration of psychiatric factors in responder and nonresponder. The cut-off values indicating the presence of psychiatric problems were defined as ≥ 10 for the doctor version and ≥ 15 for the patient version.

### Statistical analysis

Data are expressed as the mean ± the standard deviation of the mean. The Kruskal–Wallis test and the Steel–Dwass test were used to compare the NPSI scores, NPSI subscores, and BS-POP scores of the patients’ neurological symptoms and medications. The Fisher’s exact test was used to analyze differences in BS-POP scores between responders and nonresponders. A value of p < 0.05 indicated statistical significance.

### Ethics declarations

The study protocol was approved by the Human Ethics Review Committee of Fukui University Medical Faculty (Approval Number 2014046) and strictly followed the Clinical Research Guidelines of the Ministry of Health, Labor, and Welfare of the Japanese Government.

## Data Availability

Data generated and analyzed during this study are included in this published article. Data and materials are available from the corresponding author subject to reasonable request and subject to the ethical approvals in place and materials transfer agreements.
